# Development and Validation of PCR Diagnostic Assays for Detection of *Avibacterium paragallinarum* and *Ornithobacterium rhinotracheale*

**DOI:** 10.3390/vetsci11010007

**Published:** 2023-12-22

**Authors:** Ekaterina Krylova, Alexandra Bogomazova, Nataliya Kirsanova, Anastasiya Putintseva, Natalia Gorbacheva, Olga Prasolova, Irina Soltynskaya, Olga Ivanova

**Affiliations:** 1 Department of Molecular Biology, Russian State Center for Quality and Standardization of Veterinary Drugs and Feed (VGNKI), 123022 Moscow, Russia; abogomazova@rcpcm.org (A.B.); a.suhoedova@vgnki.ru (A.P.); o.prasolova@vgnki.ru (O.P.);; 2 Laboratory of Cell Biology, Lopukhin Federal Research and Clinical Center of Physical-Chemical Medicine of FMBA of Russia (Lopukhin FRCC PCM), 119435 Moscow, Russia

**Keywords:** *Ornithobacterium rhinotracheale*, ornithobacteriosis, *Avibacterium paragallinarum*, infectious coryza, analytical validation, TaqMan probes, PCR assay

## Abstract

**Simple Summary:**

Respiratory diseases cause severe economic damage to poultry farming. Common respiratory diseases in poultry include ornithobacteriosis and infectious coryza. The causative agents of these diseases, *Ornithobacterium rhinotracheale* and *Avibacterium paragallinarum*, are difficult-to-cultivate bacteria and are often found in mixed infections, which complicates the diagnosis of these diseases. PCR is a rapid, sensitive, and specific method that does not require special sample preparation; therefore, it is often used to detect infectious agents of animal diseases. We developed and validated two efficient and sensitive diagnostic assays for the rapid and accurate detection of *A. paragallinarum* and *O. rhinotracheale* DNA in bacterial isolates and clinical samples using real-time PCR. These diagnostic assays are expected to improve the differential diagnosis of respiratory diseases in poultry. PCR-based diagnostic assays can provide an alternative to microbiological cultivation and significantly reduce the time required to obtain results.

**Abstract:**

PCR is the most effective method for detecting difficult-to-cultivate pathogens and pathogens that are part of mixed infections in animals, such as *Ornithobacterium *rhinotracheale**, which causes bird ornithobacteriosis, or *Avibacterium paragallinarum*, which causes infectious coryza. In this work, we developed and validated two efficient and sensitive diagnostic assays for the rapid and accurate detection of *A. paragallinarum* and *O. rhinotracheale* DNA in bacterial isolates and clinical samples using real-time PCR with TaqMan-like probes. When designing the PCR assays, we performed in silico analysis, optimized DNA isolation methods and PCR conditions, and assessed the analytical and diagnostic performance of PCR. We designed primers and probes that have no mismatches with published whole-genome sequences of bacteria. The optimization of conditions showed that the PCR assays are sufficiently robust to changes in temperature and oligonucleotide concentration. The validation showed that the developed assays have high analytical and diagnostic sensitivity and specificity. These assays are expected to improve the differential diagnosis of respiratory diseases in chickens and turkeys.

## 1. Introduction

Respiratory diseases are among poultry’s most frequent infectious pathologies and cause serious economic damage to aviculture. They can be caused by various bacterial and viral pathogens, either independently or in association. Among the bacterial infections of the respiratory tract in poultry, infectious coryza and ornithobacteriosis occupy the leading places. The causative agent of infectious coryza in chickens, *Avibacterium paragallinarum* (formerly *Haemophilus paragallinarum*), is a Gram-negative bacterium of the *Pasteurellaceae* family; it is divided into three serogroups (A, B, and C) that comprise nine serotypes. In different countries, different serogroups and serotypes of the pathogen are predominant [1,2,3,4]. Infectious coryza of chickens is common in countries with developed aviculture, including Russia [5,6,7]; it leads to a slowdown in the growth rate of broilers and a decrease in the egg production of chickens [8]. Chickens and turkeys of all ages are susceptible to infectious coryza, especially chickens older than 4 weeks.

The causative agent of ornithobacteriosis, *Ornithobacterium rhinotracheale* (ORT), is a Gram-negative, sedentary, non-spore-forming bacterium of the *Flavobacteriaceae* family with 18 known serotypes (A–L) that are not directly associated with virulence [9,10]. Meat poultry, such as turkeys and broiler chickens, are most often affected [11,12]. Since non-specific and variable clinical manifestations characterize the disease, it is essential to have a specific and sensitive diagnostic test [13]. Phenotypic and immunological methods for the diagnosis of ornithobacteriosis still need to be developed or improved [14].

*A. paragallinarum* and *O. rhinotracheale* are difficult to cultivate in vitro, and their identification by classical microbiological methods is arduous. Moreover, isolates can be obtained from clinical material only during the acute period of the disease. These microorganisms are often accompanied by a co-infection with other pathogens such as *E. coli*, *P. multocida*, *Bordetella avium*, *Mycoplasma gallisepticum*, etc., which also complicates the isolation of pure cultures and diagnosis of the diseases [15,16]. An alternative approach to rapid diagnostics of infectious coryza and ornithobacteriosis is the use of PCR techniques. This approach does not require a large amount of material and does not require the isolation of a pure bacterial culture.

Currently, there are no validated and approved diagnostic PCR assays in the Russian Federation for detecting the DNA of *A. paragallinarum* and *O. rhinotracheale* in clinical material. Moreover, no guidelines for the PCR diagnostics of infectious coryza and ornithobacteriosis have yet been developed by the World Organization for Animal Health (WOAH, founded as OIE). Considering the growing size of the poultry industry and the economic losses from these diseases, the importance of developing PCR assays is undeniable. Thus, our work aimed to develop and validate PCR assays for the detection of pathogens in infectious coryza and ornithobacteriosis.

## 2. Materials and Methods

### 2.1. The Panel of Samples: Bacterial Isolates, Viruses, Hosts, and Clinical Samples

The panel of samples was formed, comprising isolates of *O. rhinotracheale* and *A. paragallinarum* (serogroups A, B, and C), strains of causative agents of bacterial diseases of animals and birds that are taxonomically close to the target ones, as well as causative agents of diseases with symptoms similar to infectious coryza and ornithobacteriosis, including viral ones. The strains of *O. rhinotracheale* and *A. paragallinarum* were obtained from the research collection of the Federal Center for Animal Health (ARRIAH). Table S1 presents more information about the strains. Samples of viral cDNA and genomic DNA from vaccine strains, field isolates, ATCC isolates, and animals were obtained from the research collection, which was formed in the Department of Biotechnology of the Russian State Center for Quality and Standardization of Veterinary Drugs and Feed (VGNKI). In addition, the panel included DNA samples from five avian species (*Gallus gallus*, Meleagris gallopavo, Anas platyrhynchos, Coturnix coturnix, and Columba livia), which are the primary hosts of the studied pathogens (Table S2).

Additionally, 120 clinical samples from chickens were obtained from ARRIAH. The type and number of samples are shown in Table 1. Sixty samples of pathological material were obtained from sick chickens. The diagnosis of ornithobacteriosis and infectious coryza was established based on clinical signs. Subsequent confirmation of these 60 positive clinical samples was performed through bacteriological isolation of *O. rhinotracheale* and *A. paragallinarum*, respectively. Sixty samples were also collected from healthy birds without clinical manifestations of any disease.

### 2.2. Design of Primers, Probes, and PCR Conditions

The selection of target genes was carried out using whole-genome sequences of *A. paragallinarum* and *O. rhinotracheale* deposited at NCBI (Tables S3 and S4). The selection and quality assessment of oligonucleotide primers and probes for targets was carried out using the online resources “Eurofinsgenomics PCR Primer Design” [17], “IDT OligoAnalyzer” [18], “PCR Primer Stats” [19], and “Eurofinsgenomics Oligo Analysis Tool” [20]. The optimal annealing temperature and the possibility of forming homo- and heterodimers and “hairpins” were assessed. The sequences and characteristics of the oligonucleotides are provided in Table 2 and Table 3.

An internal control (IC) whose nucleotide sequence was not homologous to any of the analyzed organisms was designed to provide confidence in successful DNA isolation and amplification. The IC sequence is presented in the Suppl. Materials. Recombinant phage λ with an inserted IC sequence was produced in GenTerra JSC (Russia).

PCR optimization was carried out on concentrations of oligonucleotides, concentration of magnesium chloride, and temperature parameters on a Rotor-Gene Q thermocycler (Qiagen, Hilden, Germany), with 10 μL of DNA per reaction volume of 25 μL (see Supplementary Materials). Optimized conditions for PCR assays for the detection of *A. paragallinarum* and *O. rhinotracheale* DNA are provided in Table 4.

### 2.3. Nucleic Acids Extraction

In this work, we tested two different methods for extracting DNA: precipitation and DNA sorption on silica gel (see Supplementary Materials).

DNA extraction was conducted from 100 μL of a bacterial suspension of pure cultures of isolates of *O. rhinotracheale* and *A. paragallinarum* (Section 2.1) using the AmpliSens^®^ RIBO-prep kit according to the manufacturer’s instructions (CRIE, Moscow, Russia).

DNA was extracted directly from clinical specimens. Preliminarily, tissue fragments were minced using scissors, diluted 1:10 in sterile 0.9% NaCl solution, homogenized for 4 min using the automated homogenizer TissueLyser LT (Qiagen, Hilden, Germany) according to the manufacturer’s instructions, and centrifuged at 1500× *g* for 2 min to obtain the supernatant. DNA was extracted from the supernatant using the AmpliSens^®^ RIBO-prep kit.

Nucleic acids from vaccine strains, field isolates, and ATCC isolates (Section 2.1) were extracted previously and stored at −40 °C in the VGNKI research collection.

Host DNA extraction was conducted from bird muscle tissue fragments using the AmpliSens^®^ DNA-sorb-C-M kit following the manufacturer’s instructions (CRIE, Moscow, Russia).

Before DNA extraction, 10 μL of IC containing 2 × 10^5^ copies/mL recombinant phages λ was added to each test tube containing the tested samples.

### 2.4. Validation of PCR Assays

Validation of diagnostic assays was carried out for compliance with the decision-making criteria for PCR methods according to a standard scheme, assessing the analytical and diagnostic characteristics of PCR [21,22,23].

### 2.5. Analytical Specificity

To confirm the specificity of the PCR assays, we used the panel of samples of bacterial isolates, viruses, and hosts described in Section 2.1. The studies were carried out in three independent rounds of PCR, each of which contained two replicates of each sample.

### 2.6. Analytical Sensitivity (Limit of Detection), Efficiency, and Coefficient of Determination (R^2^)

To determine the PCR analytical characteristics, we used DNA of plasmids containing cloned PCR fragments of the *O. rhinotracheale rnaP* and *A. paragallinarum lysS* genes and genomic DNA of *A. paragallinarum* and *O. rhinotracheale*. The recombinant pAL2-TA plasmids were produced in Evrogen (Moscow, Russia). The plasmids were sequenced to confirm the presence of the intended insert. The concentration of plasmids and genomic DNA was measured on a Quantus fluorimeter (Promega, Madison, WI, USA) following the manufacturer’s instructions. The genomic and plasmid DNA copy numbers were calculated using the following equation:

Number of copies = (X ng/µL × 6.0221 × 10^23^ molecules/mol)/ (N × 660 g/mol × 1 × 10^9^ ng/g), (1)

where X = Quantus read (ng/µL); N = length of the plasmid or bacterial genome size in bp; and 660 g/mol = average mass of one bp dsDNA.

Tenfold serial dilutions of plasmid DNA in the concentration range of 10^8^–10^2^ copies/mL were prepared. Dilutions of genomic DNA from *A. paragallinarum* and *O. rhinotracheale* in the concentration range of 10^8^ (10^7^)–10^2^ copies/mL, respectively, were prepared. To generate a standard dose-dependent curve, two replicates of each tenfold serial dilution of plasmid or genomic DNA were used, and the mean threshold cycle (Ct) value and standard deviation (SD) were calculated. The mean Ct values were plotted against log10 of DNA copy number per reaction, and linear curves were generated to estimate the curve slope, PCR efficiency, and coefficient of determination (R^2^).

PCR efficiency (E) was assessed using the standard curve slope, as shown in the following equation:

Efficiency = [10(−1/slope)] − 1] × 100,(2)

Repeatability and reproducibility were assessed by the coefficient of variability of *Ct* values, *CV*(*Ct*), and the coefficient of variability of the number of genomic copies per reaction, *CV*(*N*).

### 2.7. Diagnostic Specificity and Diagnostic Sensitivity

The diagnostic characteristics of the PCR assays were determined based on the analysis of the pathological tissues of chickens, in which the presence or absence of pathogens of infectious coryza and ornithobacteriosis was clinically confirmed. The type and number of specimens are shown in Section 2.1 (Table 1). The studies were performed in three independent rounds of PCR, each containing two replicates of each sample. Indices were calculated according to Formulas (3) and (4).

Diagnostic specificity = True negatives/(true negatives + false positives) × 100(3)

Diagnostic sensitivity = True positives/(true positives + false negatives) × 100(4)

## 3. Results

### 3.1. Design of Real-Time PCR Assays

We chose housekeeping genes with conservative sequences within species to design the PCR assays. Specifically, we chose genes that participate in translation since translation is one of the most essential processes in the cell [24]. As of February 2023, the NCBI refseq database had whole-genome data for 45 isolates of *A. paragallinarum* (Table S3). Approximately one-third of the genomes were assembled completely; the rest were at the contig or scaffold level. Among the sequenced whole-genome isolates, there were representatives of *A. paragallinarum* from all three serogroups (A, B, and C). The primers and probe used for detecting *A. paragallinarum* DNA (Table 3) were matched to the housekeeping gene *lysS*, which encodes a lysine-tRNA ligase involved in the translation process. Forward and reverse primers were designed to amplify a 139 bp segment from nt number 699,152 to 699,290 (numbering according to accession number NZ_CP050316.1). The primers were selected based on the analysis of multiple sequence alignments of the *lys* gene of *A. paragallinarum* isolates and strains from the NCBI refseq database. Figure S1A presents the annealing regions of the primers and probe, showing 100% homology between the selected oligonucleotides and all *A. paragallinarum* sequences from the NCBI refseq database.

The specificity of the oligonucleotides was assessed by aligning the selected primers and probe to the *lysS* gene sequence of *A. paragallinarum*-related bacteria (Figure S1B). The oligonucleotides have from two to six mismatches with the sequence of the *lysS* gene of the closest relatives. Thus, PCR with the selected primers and probe should specifically detect the DNA of *A. paragallinarum*.

For the PCR detection of *O. rhinotracheale*, we selected primers and a probe (Table 3) for a region of the *rnaP* gene (ribozyme RNase P gene), which is highly conserved in this species. The target fragment is 101 bp long, from nt number 72,116 to 72,216 (numbering according to accession number NZ_CP006828.1). The *rnaP* gene is a housekeeping gene that belongs to the category of non-coding RNA genes. The sequence of this gene consists of sub-sequences that are highly conservative among bacteria and sub-sequences that are specific to more narrow taxonomic groups [25]. The primers were selected by analyzing multiple sequence alignments of the *rnaP* gene of all isolates and strains of *O. rhinotracheale*, whose whole-genome sequences were deposited in the NCBI refseq database. Figure S2A shows the annealing region of the selected primers and probe. The forward primer at positions 5 and 17 has mismatches in the sequence with the genome of the isolate FARPER-174b (NZ_CP CP035107.1). We decided to ignore this since this isolate was isolated from the discharge of the paranasal sinuses of laying hens in Peru, i.e., in an agrarian region remote from Russia.

In silico, the primer specificity was assessed using the Primer-BLAST online resource. Figure S2B shows multiple alignment in the region of primer and probe annealing in the *rnaP* gene sequence taken from the representative genome of *O. rhinotracheale* and representative genomes of some related species with a high similarity in the *rnaP* gene (*Ornithobacterium hominis*, *Riemerella anatipestifer*, *Bergeyella cardium*, and *Capnocytophaga gingivalis*). The primers and probe have many mismatches with the *rnaP* gene sequence of other bacterial species. Thus, PCR with the selected primers and probe should effectively detect the DNA of *O. rhinotracheale*.

### 3.2. Analytical Specificity

To confirm the specificity of the PCR assays, a panel of DNA samples was used. The PCR results are presented in Table 5.

In our sample panel, the PCR assays detected the pathogens of infectious coryza and ornithobacteriosis with 100% specificity. DNA amplification was observed only for the target microorganisms.

### 3.3. Analytical Sensitivity (Limit of Detection), Efficiency, and Coefficient of Determination (R^2^)

In the experiments to determine analytical characteristics, it became apparent that the LOD of detection for *A. paragallinarum* was 7 × 10^3^ copies/mL, and for *O. rhinotracheale* it was 4 × 10^3^ copies/mL. The efficiency, slope, and coefficient of determination for PCR detection of *A. paragallinarum* DNA and *O. rhinotracheale* DNA corresponded to the accepted criteria (efficiency > 90%, slope 3.3–3.6, R^2^ > 0.98) (Table 6 and Table 7).

### 3.4. Repeatability

We determined repeatability using PCR with plasmid and genomic DNA, whose copy numbers per reaction were close to the limit of PCR sensitivity. PCR with 70–170 copies of DNA from *A. paragallinarum* per reaction (N) was performed in 10 replicates along with PCR with calibration samples of plasmid or genomic DNA serially diluted from 10^8^ to 10^4^ copies/mL. PCR with 40–105 copies of DNA from *O. rhinotracheale* per reaction (N) was performed in 10 replicates along with PCR with calibration samples of plasmid DNA serially diluted from 10^8^ to 10^4^ copies/mL or genomic DNA serially diluted from 10^7^ to 10^4^ copies/mL. After that, *CV*(*Ct*) and *CV*(*N*) were calculated. The results are presented in Table 8.

For both PCR assays, good values of Ct repeatability were obtained; the coefficient of variability *CV*(*Ct*) ranged from 0.5% to 1.4%. The coefficient of variability for DNA copies per reaction *CV*(*N*) ranged from 10.7% to 25%, which corresponds to the accepted criteria.

Thus, ~70 copies of the *A. paragallinarum lysS* gene and ~40 copies of the *O. rhinotracheale rnaP* gene were reproducibly detected in the respective PCR assays. In other words, the LOD of the PCR assay for *A. paragallinarum* was 70 genomic copies per reaction (=7 × 10^3^ copies/mL), and for *O. rhinotracheale* it was 42 genomic copies per reaction (=4.2 × 10^3^ copies/mL) (Table 6 and Table 7).

To obtain reproducible results in laboratory practice, samples with target DNA content above the LOD should be considered “positive”. This requires setting the cutoff limit of the cycle. We considered the cutoff cycle limit at Ct ≤ 33 for the PCR assay for *A. paragallinarum* detection and at Ct ≤ 32 for the PCR assay for *O. rhinotracheale* detection.

### 3.5. Reproducibility

We assessed PCR reproducibility using three independent experiments performed on different days by different operators using different thermal cyclers. In each experiment, we used two replicates of each tenfold serial dilution of plasmid DNA and genomic DNA, as well as DNA samples diluted close to the PCR sensitivity limit. The results are presented in Table 9 and Table 10.

For the *A. paragallinarum* PCR assay, the coefficient of variability *CV*(*Ct*) ranged from 0.3 to 1.4% and the *CV*(*N*) from 1.9% to 20.7%. For the *O. rhinotracheale* PCR assay, the coefficient of variability *CV*(*Ct*) ranged from 0.4% to 1.9% and the *CV*(*N*) from 2.2% to 22.7%. These values indicate acceptable reproducibility for both PCR assays.

### 3.6. Diagnostic Specificity and Sensitivity

To assess the diagnostic characteristics of the PCR assays, 30 known positive and known negative clinical samples from chickens were analyzed for each pathogen separately. The results are presented in Tables S5 and S6. All known positive samples containing *A. paragallinarum* or *O. rhinotracheale* were found to contain DNA of *A. paragallinarum* or *O. rhinotracheale*. All known negative samples that did not contain *A. paragallinarum* or *O. rhinotracheale* tested negative. The indices of diagnostic sensitivity and specificity calculated from the obtained results with a 95% confidence level are presented in Table 11.

## 4. Discussion

The common avian bacterial pathogens, A. *paragallinarum and O. rhinotracheale,* are challenging to diagnose. Not surprisingly, the first PCR-based test for A. *paragallinarum* identification was developed in the 1990s. To identify *A. paragallinarum* DNA, Chen et al. developed a PCR-based assay with detection using gel electrophoresis in 1996. The primers were selected for the region of the *pyrG* gene that encodes a glutamine-hydrolyzing enzyme [26]. In 2008, Corney et al. developed another assay for the detection of *A. paragallinarum* DNA based on real-time PCR with primers for the *pyrG* gene [27]. Clothier et al. successfully validated this assay as a diagnostic one in 2019 [28]. In 2021, Kuchipudi et al. [29] developed an assay based on real-time PCR with primers for a region of the *A. paragallinarum recN* gene encoding a repair protein. During validation, the assay was more sensitive compared to the assay of Corney et al. [27]. Bogomazova et al. [30] found that the primers perfectly matched *recN* gene sequences from all 50 *A. paragallinarum* genomes deposited in the NCBI refseq database. However, the recN gene is not part of the minimal set of genes required for the bacterial cell [24], and its loss may be compatible with the preservation of viability.

To detect *O. rhinotracheale* DNA, a real-time PCR assay with primers for the 16S ribosomal RNA gene (16S rRNA) was developed in 2013 [31]. In 2022, Hashish et al. improved this technique by changing the probe position and proposing their own unique set of primers and probe for the same target [32]. It should be noted that the 16S rRNA gene is traditionally used for bacterial identification and phylogenic studies; it belongs to the category of non-coding RNA genes, is conservative, and has multiple copies [33]. The genome of *O. rhinotracheale* contains three copies of the 16S rRNA gene. We tested the design of Hashish et al. [32] on all 16 *O. rhinotracheale* whole genomes deposited in the NCBI database and found that in 14 out of 16 cases (88%), the primers and probe annealed perfectly to the 16S rRNA gene sequence. However, in 2 strains out of 16, we found 1–2 mismatches between the 16S rRNA gene sequence and the probe sequence, which can significantly reduce PCR efficiency.

Bogomazova A. et al. [30] described several principles for in silico analysis in the design of PCR assays for the diagnosis of infectious animal diseases. Namely, it is necessary to (1) take into account the intraspecies genetic polymorphism of the bacterial pathogen; (2) indicate the NCBI database (nt/nr or refseq genomes or wgs) for BLAST searches and the number of bacterial gene sequences available for analysis; and (3) indicate the presence or absence of polymorphism at the sites of primer and probe annealing. The design of the assays for identifying pathogens of infectious coryza and ornithobacteriosis in this work was carried out considering these principles. As target genes for PCR, we chose genes participating in translation since these genes are part of a minimal gene set essential for bacterial viability [24]. This approach should provide the necessary robustness to the PCR assays developed.

## 5. Conclusions

In this work, we developed and validated two efficient and sensitive diagnostic assays for the rapid and accurate detection of *A. paragallinarum* and *O. rhinotracheale* DNA, both in bacterial isolates and in clinical samples, using real-time PCR with TaqMan-like probes. The use of these diagnostic assays will improve the differential diagnosis of respiratory diseases in chickens and turkeys. PCR-based diagnostic assays can be considered an alternative to microbiological culturing and significantly reduce the time required to obtain results.

## Figures and Tables

**Table 1:** Clinical samples of *A. paragallinarum* and *O. rhinotracheale*.

Pathogen	Clinical Sample Type	Number	
		Known Positives	Known Negatives
*A. paragallinarum*	fragments of chicken beaks and sinuses	30	30
*O. rhinotracheale*	fragments of chicken trachea	30	30

**Table 2:** Characteristics of targets and oligonucleotides for the PCR assays for detecting *A. paragallinarum* DNA.

Pathogen	Target Gene	Gene Description	Oligo	Sequence (5′–3′)	Length, bp	Amplicon Size, bp
*Avibacterium paragallinarum*	*lysS*	Lysine-tRNA ligase	LysS-F ^1^	GTGAAAGAGGATCTGTACGAC	21	139
			LysS-P ^2^	ROX– TCGATCGTGCCAAAGCCTTGG–BHQ2	21	
			LysS-R ^3^	GCTGAATTAAGTGATGTTCTGC	22	
Internal control	synthetic construct		IC-F	GTGCGATGGTCCGACTTAT	19	90
			IC-P	R6G–CTAGCTGGGCGTCAGGAATCC–BHQ1	21	
			IC-R	GGTCAGTTATTTACCTACGACAG	23	

^1^ F—forward primer; ^2^ P—probe; ^3^ R—reverse primer.

**Table 3:** Characteristics of targets and oligonucleotides for the PCR assays for detecting *O. rhinotracheale* DNA.

Pathogen	Target Gene	Gene Description	Oligo	Sequence (5′–3′)	Length, bp	Amplicon Size, bp
*Ornithobacterium rhinotracheale*	*rnaP*	DNA- directed RNA polymerase	RnaP-F ^1^	TAACACCCGCCACTTGTTT	19	101
			RnaP-P ^2^	ROX– TTCTGCTGCACTAGTCCTCCTT GC–BHQ2	24	
			RnaP-R ^3^	CCACGCAGAGTTTACCTGATT	21	
Internal control	synthetic construct		IC-F	GTGCGATGGTCCGACTTAT	19	90
			IC-P	R6G–CTAGCTGGGCGTCAGGAATCC–BHQ1	21	
			IC-R	GGTCAGTTATTTACCTACGACAG	23	

^1^ F—forward primer; ^2^ P—probe; ^3^ R—reverse primer.

**Table 4:** PCR conditions.

Pathogen	Target Gene	F/R/P, nM	MgCl_2_, mM	PCR Program
*Ornithobacterium rhinotracheale*	*rnaP*	240/240/120	2.5	95 °C for 15 min, 40 cycles of 95 °C for 15 s, and 62 °C for 45 s
Internal control	synthetic construct	120/120/60		
*Avibacterium paragallinarum*	*lysS*	240/240/120	2.5	95 °C for 15 min, 40 cycles of 95 °C for 15 s, and 62 °C for 45 s
Internal control	synthetic construct	120/120/60		

**Table 5:** Results of PCR for detection of *A. paragallinarum lysS* and *O. rhinotracheale rnaP* gene fragments.

No.	Organism	Sample Type	Description	Mean Ct	
				*rnaP*	*lysS*
1	*A.paragallinarum* ser. B	bacterial suspension	collection strain 1116	-	14.23
2	*A.paragallinarum* ser. B	bacterial suspension	collection strain 1818	-	17.93
3	*A.paragallinarum* ser. B	bacterial suspension	collection strain 5111	-	18.54
4	*A.paragallinarum* ser. A	bacterial suspension	collection strain 6261	-	14.77
5	*A.paragallinarum* ser. A	bacterial suspension	ATCC 29545	-	17.41
6	*A.paragallinarum* ser. C	bacterial suspension	collection strain 1919	-	18.98
7	*A.paragallinarum*	bacterial suspension	collection strain Kostroma	-	30.12
8	*O.rhinotracheale*	bacterial suspension	collection strain OR-21	22.36	-
9	Avian encephalomyelitis	viral cDNA	vaccine strain Calnec 1143 M	-	-
10	Avian metapneumovirus	viral cDNA	vaccine strain TRT 11/94 ser. B;	-	-
11	Avian metapneumovirus	viral cDNA	vaccine strain Clone K ser. A	-	-
12	Avian metapneumovirus	viral cDNA	vaccine strain PV03-B	-	-
13	Infectious laryngotracheitis virus	viral gDNA	vaccine strain CHP 50	-	-
14	Avian poxvirus	viral gDNA	vaccine strain KEM-7	-	-
15	Chicken anemia virus	viral gDNA	vaccine strain Cux-1	-	-
16	Infectious bronchitis virus	viral cDNA	vaccine strain H120 ser Massachusetts	-	-
17	Infectious bursal disease virus	viral cDNA	vaccine strain Winterfield 2512	-	-
18	Newcastle virus	viral cDNA	vaccine strain La-Sota	-	-
19	Egg drop syndrome virus	viral gDNA	vaccine strain EDS-76 B-93	-	-
20	Influenza virus type A	viral cDNA	vaccine strain Chicken/USSR/315/70	-	-
21	Influenza virus type A H14N6	viral cDNA	vaccine strain Mallard/Astrakhan 263/82	-	-
22	Turkey herpes virus	viral gDNA	field isolate	-	-
23	Avian reovirus	viral cDNA	field isolate	-	-
24	*Mycoplasma gallisepticum*	bacterial gDNA	field isolate	-	-
25	*Salmonella* Typhimurium	bacterial gDNA	ATCC 14028	-	-
26	*Staphylococcus aureus*	bacterial gDNA	ATCC 39591	-	-
27	*Streptococcus* sp.	bacterial gDNA	field isolate	-	-
28	*Mannhemia haemolytica*	bacterial gDNA	field isolate	-	-
29	*Arcanobacterium pyogenes*	bacterial gDNA	ATCC 8164	-	-
30	*Pasterella multocida*	bacterial gDNA	field isolate	-	-
31	*Glaesserella parasuis*	bacterial gDNA	ATCC 19417	-	-
32	*Mycobacterium avium*	bacterial gDNA	field isolate	-	-
33	*Enterococcus avium*	bacterial gDNA	ATCC 14025	-	-
34	*Escherichia coli*	bacterial gDNA	ATCC 25922	-	-
35	*Aspergillus brasiliensis*	bacterial gDNA	ATCC 16404	-	-
36	*Bordetella bronchiseptica*	bacterial gDNA	ATCC 10580	-	-
37	*Histophilus somni*	bacterial gDNA	field isolate	-	-
38	*Gallus gallus*	gDNA	isolated from muscle tissue	-	-
39	*Meleagris gallopavo*	gDNA	isolated from muscle tissue	-	-
40	*Anas platyrhynchos*	gDNA	isolated from muscle tissue	-	-
41	*Coturnix coturnix*	gDNA	isolated from muscle tissue	-	-
42	*Columba livia*	gDNA	isolated from muscle tissue	-	-
43	Internal control	phage λ gDNA	recombinant phage λ	-	-

**Table 6:** Limit of detection and standard curve parameters of PCR assay for *A. paragallinarum.*

DNA Type	Target Gene	Serotype	LOD	Linear Equation	R^2^	Efficiency
plasmid with *A. paragallinarum lysS* fragment	*lysS*	nd	7000 copies/mL	y = −3.349x + 37.201	0.999	99%
*A. paragallinarum* genomic DNA		A		y = −3.336x + 38.510	0.996	99%
		B		y = −3.464x + 38.615	0.999	94%
		C		y = −3.413x + 37.668	0.999	96%

**Table 7:** Limit of detection and standard curve parameters of PCR assay for *O. rhinotracheale.*

DNA Type	Target Gene	LOD	Linear Equation	R^2^	Efficiency
plasmid with *O. rhinotracheale rnaP* fragment	*rnaP*	4000 copies/mL	y = −3.362x + 38.448	0.999	98%
*O. rhinotracheale* OR21 genomic DNA			y = −3.308x + 37.729	0.999	101%

**Table 8:** Assessment of repeatability of PCR assays.

DNA Type	Copy Number/PCR	Replicates	Mean *Ct*	*CV*(*Ct*), %	Mean Copy Number/ PCR (N)	*CV*(*N*), %
plasmid with *A. paragallinarum lysS* fragment	170	10	29.83	0.8	223.3	16.9
	85	10	30.78	1.4	120.5	24.6
genomic DNA of *A. paragallinarum*, ser. B	143	10	32.10	0.8	141.2	17.4
	70	10	33.14	1.4	74.2	24.1
plasmid with *O. rhinotracheale rnaP* fragment	105	10	31.52	0.7	93.2	14.6
	42	10	32.16	0.5	60.3	11.1
genomic DNA of *O. rhinotracheale* OR21	92	10	31.79	0.5	107.9	10.0
	46	10	32.78	1.0	58.1	20.7

**Table 9:** Assessment of reproducibility of the PCR assay for *A. paragallinarum* detection.

DNA Type	Copy Number /PCR	Mean *Ct*	*CV*(*Ct*), %	Mean Copy Number/PCR (N)	*CV*(*N*), %
plasmid with *A. paragallinarum lysS* fragment	1.7 × 10^6^	16.55	1.4	1,714,465	5.8
	1.7 × 10^5^	19.97	0.8	166,931	5.7
	1.7 × 10^4^	23.30	0.9	17,067	1.9
	1.7 × 10^3^	26.65	0.5	1731	7.6
	1.7 × 10^2^	29.97	1.1	179	7.3
	85	31.02	1.2	102	20.7
genomic DNA of *A. paragallinarum* ser. B	5.7 × 10^6^	15.19	1.1	5,632,822	4.1
	5.7 × 10^5^	18.56	1.1	572,344	3.7
	5.7 × 10^4^	21.96	0.7	56,599	3.6
	5.7 × 10^3^	25.29	0.8	5915	5.0
	5.7 × 10^2^	28.72	0.3	575	14.0
	70	32.97	0.7	72	15.3

**Table 10:** Assessment of reproducibility of the PCR assay for *O. rhinotracheale* detection.

DNA Type	Copy Number/PCR	Mean *Ct*	*CV*(*Ct*), %	Mean Copy Number/PCR (N)	*CV*(*N*), %
plasmid with *O. rhinotracheale rna P* fragment	4.2 × 10^6^	15.97	1.1	4,839,048	10.6
	4.2 × 10^5^	19.41	0.8	431,128	6.9
	4.2 × 10^4^	22.90	0.4	37,328	14.7
	4.2 × 10^3^	26.10	0.4	3965	14.0
	4.2 × 10^2^	29.48	1.0	369	9.0
	42	32.54	1.6	44	22.7
genomic DNA of *O. rhinotracheale OR21*	1.8 × 10^5^	20.14	1.2	203,107	6.5
	1.8 × 10^4^	24.08	1.3	16,098	7.7
	1.8 × 10^3^	27.51	1.5	1748	2.2
	1.8 × 10^2^	30.89	1.9	199	13.2
	46	32.85	1.1	55	19.2

**Table 11:** Diagnostic specificity and diagnostic sensitivity of the PCR assays for *A. paragallinarum* and *O. rhinotracheale* DNA detection.

Pathogen	Diagnostic Sensitivity (95% Confidence Level)	Diagnostic Specificity (95% Confidence Level)
*O. rhinotracheale*	100% (88.4–100%)	100% (88.4–100%)
*A. paragallinarum*	100% (88.4–100%)	100% (88.4–100%)

## Data Availability

Data are contained within the article and Supplementary Materials.

## Supplementary Materials

The following supporting information can be downloaded at: https://www.mdpi.com/article/10.3390/vetsci11010007/s1, Table S1: The sample panel of bacterial isolates from the research collection of ARRIAH; Figure S1: Multiple alignment of *lysS* gene sequences in the region of annealing of primers and probe; Table S2: The DNA sample panel from the research collection of the Department of Biotechnology at VGNKI; Figure S2: Multiple alignment of *rnaP* gene sequences in the region of annealing of primers and probe; Table S3: List of whole-genome sequences of *A. paragallinarum* isolates in the NCBI refseq database [34,35,36,37,38,39,40,41,42,43,44,45]; Table S4: List of whole-genome sequences of *O. rhinotracheale* isolates in the NCBI refseq database [46,47,48]; Table S5: Test results for clinical samples of *A paragallinarum* (A.pg) (fragments of chicken beaks and sinuses); Table S6: Test results for clinical samples of *O. rhinotracheale* (ORT) (chicken tracheal homogenate).
