# Supplementary material for: Development and Validation of PCR Diagnostic Assays for Detection of Avibacterium paragallinarum and Ornithobacterium rhinotracheale
Source: Vet Sci. 2023 Dec 22;11(1):7. doi: 10.3390/vetsci11010007 (PMC10819020; doi:10.3390/vetsci11010007)
Supplement: Supplementary file 1 [file vetsci-11-00007-s001.zip › vetsci-2695919-supplementary.pdf]

Development and validation of PCR diagnostic assays for detection of *Avibacterium paragallinarum* and *Ornithobacterium rhinotracheale*

Supplementary materials:

Table S1: The sample panel of bacterial isolates from the research collection of ARRIAH.

Organism	Attributes				
	collection date	host	host disease	isolation source	geographic location
A.paragallinarum ser. B 1116	2017	chicken	infectious coryza	infraorbital sinus	Russia, Republic of Mordovia
A.paragallinarum ser. B 1818	2017	chicken	infectious coryza	infraorbital sinus	Belarus
A.paragallinarum ser. B 5111	2017	chicken	infectious coryza	infraorbital sinus	Russia, Orenburg region
A.paragallinarum ser. A 6261	2018	chicken	infectious coryza	infraorbital sinus	Russia, Vladimir region
A.paragallinarum ser. A, ATCC 29545	2019	chicken	infectious coryza	infraorbital sinus	Germany
A.paragallinarum ser. C 1919	2018	chicken	infectious coryza	infraorbital sinus	Russia, Republic of Mordovia
A.paragallinarum Kostroma	2018	chicken	infectious coryza	infraorbital sinus	Russia, Kostroma region
O.rhinotracheale OR-21	2021	turkey	ornithobacteri osis	trachea	Russia, Penza region

Table S2: The DNA sample panel from the research collection of the Department of Biotechnology at VGNKI.

**Organism**	**Sample type**	**Description**
Avian Encephalomyelitis	viral cDNA	vaccine strain Calnec 1143M
Avian metapneumovirus	viral cDNA	vaccine strain TRT 11/94 ser. B
Avian metapneumovirus	viral cDNA	vaccine strain Clone K ser. A
Avian metapneumovirus	viral cDNA	vaccine strain PV03-B
Infectious laryngotracheitis virus	viral gDNA	vaccine strain CHP 50
Avian poxvirus	viral gDNA	vaccine strain KEM-7
Chicken anemia virus	viral gDNA	vaccine strain Cux-1
Infectious bronchitis virus	viral cDNA	vaccine strain H120 ser Massachusetts
Infectious bursal disease virus	viral cDNA	vaccine strain Winterfield 2512
Newcastle virus	viral cDNA	vaccine strain La-Sota
Egg drop syndrome virus	viral gDNA	vaccine strain EDS-76 B-93
Influenza virus type A	viral cDNA	vaccine strain Chicken/USSR/315/70
Influenza virus type A H14N6	viral cDNA	vaccine strain Mallard/Astrakhan 263/82
Turkey herpes virus	viral gDNA	field isolate
Avian reovirus	viral cDNA	field isolate
Mycoplasma gallisepticum	bacterial gDNA	field isolate
Salmonella Typhimurium	bacterial gDNA	ATCC 14028
Staphylococcus aureus	bacterial gDNA	ATCC 39591
Streptococcus sp.	bacterial gDNA	field isolate
Mannhemia haemolytica	bacterial gDNA	field isolate

Organism	Sample type	Description
Arcanobacterium pyogenes	bacterial gDNA	ATCC 8164
Pasterella multocida	bacterial gDNA	field isolate
Glaesserella parasuis	bacterial gDNA	ATCC 19417
Mycobacterium avium	bacterial gDNA	field isolate
Enterococcus avium	bacterial gDNA	ATCC 14025
Escherichia coli	bacterial gDNA	ATCC 25922
Aspergillus brasiliensis	bacterial gDNA	ATCC 16404
Bordetella bronchiseptica	bacterial gDNA	ATCC 10580
Histophilus somni	bacterial gDNA	field isolate
Gallus gallus	gDNA	isolated from muscle tissue
Meleagris gallopavo	gDNA	isolated from muscle tissue
Anas platyrhynchos	gDNA	isolated from muscle tissue
Coturnix coturnix	gDNA	isolated from muscle tissue
Columba livia	gDNA	isolated from muscle tissue
Internal control	phage λ gDNA	recombinant phage λ (GenTerra JSC, Russia)

Nucleotide sequence of internal control

> IC
GTGCGATGGTCCGACTTATTCGTAGAGGGCTAGCTGGGCGTCAGGAATCCCAGGTGGAGGGTGT
GTCCTGTCGTAGGTAAATAACTGACC

PCR optimization

PCR optimization was carried out based on concentrations of oligonucleotides, concentration of magnesium chloride, and temperature parameters. Tenfold serial dilutions of plasmid DNA containing target fragments of the *O. rhinotracheale rnaP* and the *A. paragallinarum lysS* genes or genomic DNA of *A paragallinarum* and *O. rhinotracheale* were used. Three independent experiments were carried out on different days on different thermal cyclers. In each experiment, two replicates of each tenfold serial dilution of plasmid or genomic DNA were used, and the mean threshold cycle Ct value and standard deviation (SD) were calculated (data not shown).

To optimize the concentration of oligonucleotides, four master mixes were prepared with different concentrations of primers and probes within the working range of 200–1,000 nM (F/R/P): 120/120/60 nM, 240/240/120 nM, 480/480/240 nM, and 960/960/480 nM. Since the sensitivity and efficiency of the PCR assay at different concentrations of oligonucleotides changed insignificantly, the following concentrations were chosen: for the detection of *A. paragallinarum* DNA: 240/ 240/ 120 nM; for the detection of *O. rhinotracheale* DNA: 240/ 240/ 120 nM; and for the internal control: 120/ 120/ 60 nM.

To optimize the concentration of Mg^{2+} ions, three working mixtures were prepared within the range of 1–4 mM: 1.5 mM, 2.5 mM, and 3.5 mM. At Mg^{2+} concentrations of 2.5 mM and 3.5 mM, the lowest values of Ct and the highest sensitivity were recorded. When the Mg^{2+} concentration was reduced to 1.5 mM, PCR assay sensitivity decreased, and in some experiments, PCR efficiency was lower than the established criteria. Thus, the optimal Mg^{2+} concentration was found to be 2.5 mM.

To optimize the annealing temperature, studies were carried out at 62 °C, calculated in accordance with the melting temperatures of the primers and probes, as well as at 60 °C and 64 °C, which were 2 °C higher and lower than the calculated melting temperature, respectively. At all tested annealing temperatures, similar values of Ct were observed on both plasmid and genomic DNA. Based on the results obtained, as well as taking into account an increased risk of non-specific annealing with decreasing temperature, the optimal annealing temperature was chosen to be 62 °C.

Moreover, when testing various concentrations of oligonucleotides and magnesium chloride as well as annealing temperatures, the PCR assays for infectious coryza and ornithobacteriosis showed 100% specificity for the panel of samples described above (see Materials and Methods). Only the amplification of the DNA of the target microorganisms was observed.

DNA extraction

In this work, we tested two different methods for extracting DNA from *A. paragallinarum* and *O. rhinotracheale* across different types of samples (isolates of *A. paragallinarum* and *O. rhinotracheale* and pathological material from chickens, as detailed in Section 2.1). These methods involved precipitation using the AmpliSens® RIBO-prep kit and DNA sorption on silica gel using the AmpliSens® DNA-sorb-B kit (both CRIE, Russia) in accordance with the manufacturer's instructions. According to the results obtained, both tested methods can be used to isolate DNA from samples of pathological tissues from birds and bacterial isolates for the purpose of detecting the DNA of the target pathogen using real-time PCR. However, more optimal indices of threshold cycles (Ct) (and, accordingly, extraction efficiency) were obtained using the precipitation-based method (data not shown).

Table S3: List of whole-genome sequences of *A. paragallinarum* isolates in the NCBI refseq database. The genome used in the NCBI database as a representative genome is highlighted in bold italics.

No.	Isolate	Serotype	Assembly	Level	Size, (Mb)	GC, %	Reference
1	JF4211	A	GCA_000442905.1	Contig	2.87	41.1	[34]
2	NCTC11296	nd	GCA_900450705.1	Contig	2.86	41.1	[35]
3	NCTC10926	nd	GCA_900445345.1	Contig	2.79	41.2	
4	221	A	GCA_000348525.1	Contig	2.67	41	[36]
5	***ESV-135***	C-1	***GCA_011765605.1***	***Complete***	***2.52***	***40.9***	[37]
6	AVPG2015	nd	GCA_000969315.2	Complete	2.53	40.9	
7	CL	C	GCA_000969305.1	Contig	2.41	41.3	
8	Modesto	C	GCA_004931865.1	Contig	2.35	40.9	[38]
9	SCPM-O-B-8407	nd	GCA_003204295.1	Contig	2.55	40.8	nd
10	SCPM-O-B-8406	nd	GCA_003204245.1	Contig	2.56	40.8	nd
11	CCUG 12835	nd	GCA_002921155.1	Contig	2.46	41	[39]
12	AVPG 221	A	GCA_004212415.1	Contig	2.66	41.1	nd
13	Modesto	C	GCA_004212375.1	Contig	2.50	40.9	nd
14	SA-3	C	GCA_004212355.1	Contig	2.39	41	nd
15	2671	B	GCA_004212345.1	Contig	2.34	41	nd
16	FARPER-174	C	GCA_003864155.1	Complete	2.43	40.9	[40]
17	Hp8	A	GCA_008633115.1	Scaffold	2.36	41	[41]
18	ADL-AP02	nd	GCA_012850815.1	Complete	2.42	40.9	[42]
19	ADL-AP07	nd	GCA_012850655.1	Complete	2.42	40.9	
20	ADL-AP15	nd	GCA_012850275.1	Complete	2.42	40.9	
21	ADL-AP16	nd	GCA_012850075.1	Complete	2.42	40.9	
22	ADL-AP17	nd	GCA_012849875.1	Complete	2.42	40.9	
23	ADL-AP10	nd	GCA_012850455.1	Complete	2.42	40.9	
24	ADL-AP01	nd	GCA_012850955.1	Complete	2.42	40.9	
25	72	C	GCA_000221945.4	Contig	2.45	40.8	[43]
26	APX1-1S-1	nd	GCA_015354985.1	Scaffold	2.59	40.8	[44]
27	AP1-3S-1	nd	GCA_015355095.1	Scaffold	2.59	40.8	
28	AP12-4N-1	nd	GCA_015354995.1	Scaffold	2.59	40.8	
29	APX1-2N-1	nd	GCA_015354975.1	Scaffold	2.59	40.8	

No.	Isolate	Serotype	Assembly	Level	Size, (Mb)	GC, %	Reference
30	AP1-1N-1	nd	GCA_015355125.1	Scaffold	2.59	40.8	
31	Z2S-1-2	nd	GCA_015354775.1	Scaffold	2.59	40.8	
32	Z1N-2-2	nd	GCA_015354865.1	Scaffold	2.59	40.8	
33	APX3-2S-2	nd	GCA_015354895.1	Scaffold	2.59	40.8	
34	APX1-1N-2	nd	GCA_015354935.1	Scaffold	2.59	40.8	
35	APX2-2N-2	nd	GCA_015354955.1	Scaffold	2.59	40.8	
36	AP12-5S-1	nd	GCA_015355035.1	Scaffold	2.59	40.8	
37	AP1-2N-1	nd	GCA_015355065.1	Scaffold	2.59	40.8	
38	Y2S-2	nd	GCA_015354885.1	Scaffold	2.56	40.8	
39	AP1-3N-1	nd	GCA_015355055.1	Scaffold	2.59	40.8	
40	Z1N-2-1	nd	GCA_015354815.1	Scaffold	2.59	40.8	
41	Z1N-1-2	nd	GCA_015354845.1	Scaffold	2.59	40.8	
42	p4chr1	A	GCA_019822885.1	Complete	2.77	41	[45]
43	omv-p4chr1	nd	GCA_020523945.1	Contig	2.69	40.9	nd
44	ZJ-C	nd	GCA_022964995.1	Complete	2.68	40.9	nd
45	M	nd	GCA_020892835.1	Complete	2.77	41	nd

A

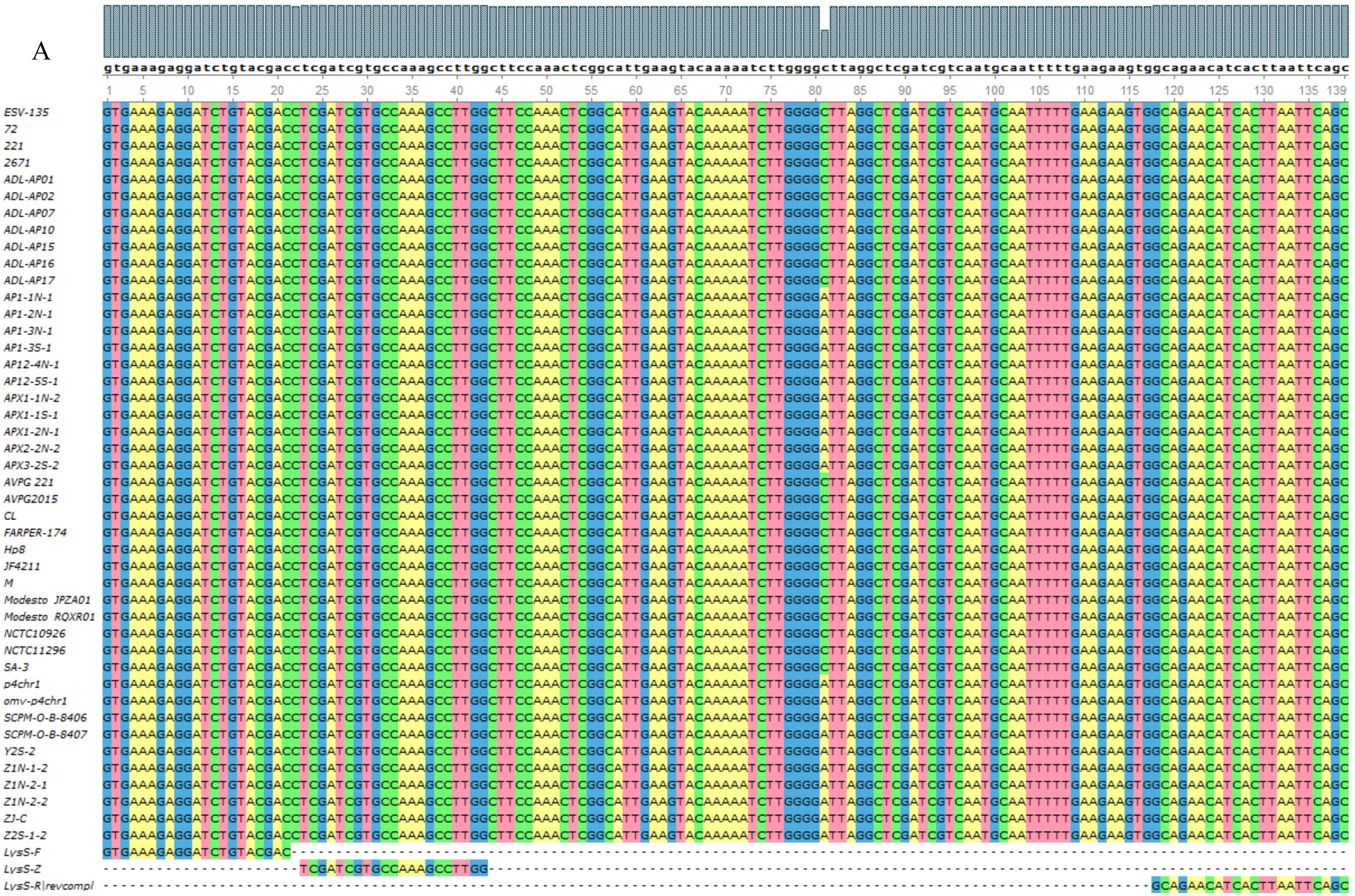

B

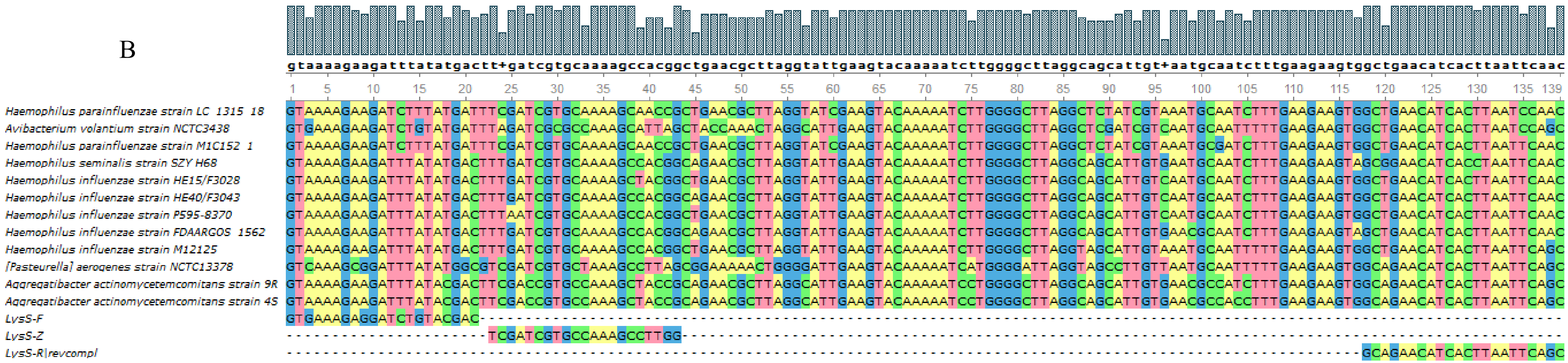

Figure S1: Multiple alignment of *lysS* gene sequences in the region of annealing of primers and probe.
A) *A. paragallinarum;* B) related bacteria.
The reverse primer sequence is given in the reverse-complement orientation.

Table S4: List of whole-genome sequences of *O. rhinotracheale* isolates in the NCBI refseq database. The genome used in the NCBI database as a representative genome is highlighted in bold italics.

No.	Strain	Assembly	Level	Size, (Mb)	GC%	Reference
1	***ORT-UMN 88***	***GCA_000756505.1***	***Complete***	***2.40***	***37.50***	[46]
2	IRI-Sh	GCA_024722395.1	Complete	2.40	37.50	nd
3	FARPER-174b	GCA_004088395.1	Complete	2.25	38.30	nd
4	OR14	GCA_023016705.1	Contig	2.46	37.70	nd
5	46	GCA_023016765.1	Contig	2.37	37.50	nd
6	LMG 18861	GCA_009659645.1	Contig	2.35	37.30	[47]
7	LMG 18856	GCA_009659665.1	Contig	2.42	37.10	
8	LMG 19032	GCA_009659705.1	Contig	2.41	37.10	
9	C183	GCA_023016535.1	Contig	2.42	37.40	nd
10	J209	GCA_023016785.1	Contig	2.60	37.60	nd
11	ORT3331-2017	GCA_017084005.1	Scaffold	2.30	34.30	nd
12	97	GCA_022832875.1	Complete	2.42	37.40	nd
13	A171	GCA_022832855.1	Complete	2.41	37.50	nd
14	K223	GCA_022832975.1	Complete	2.42	37.39	nd
15	DSM 15997	GCA_000265465.1	Complete	2.40	37.20	[48]
16	H06-030791	GCA_000764515.1	Contig	2.32	37.20	nd

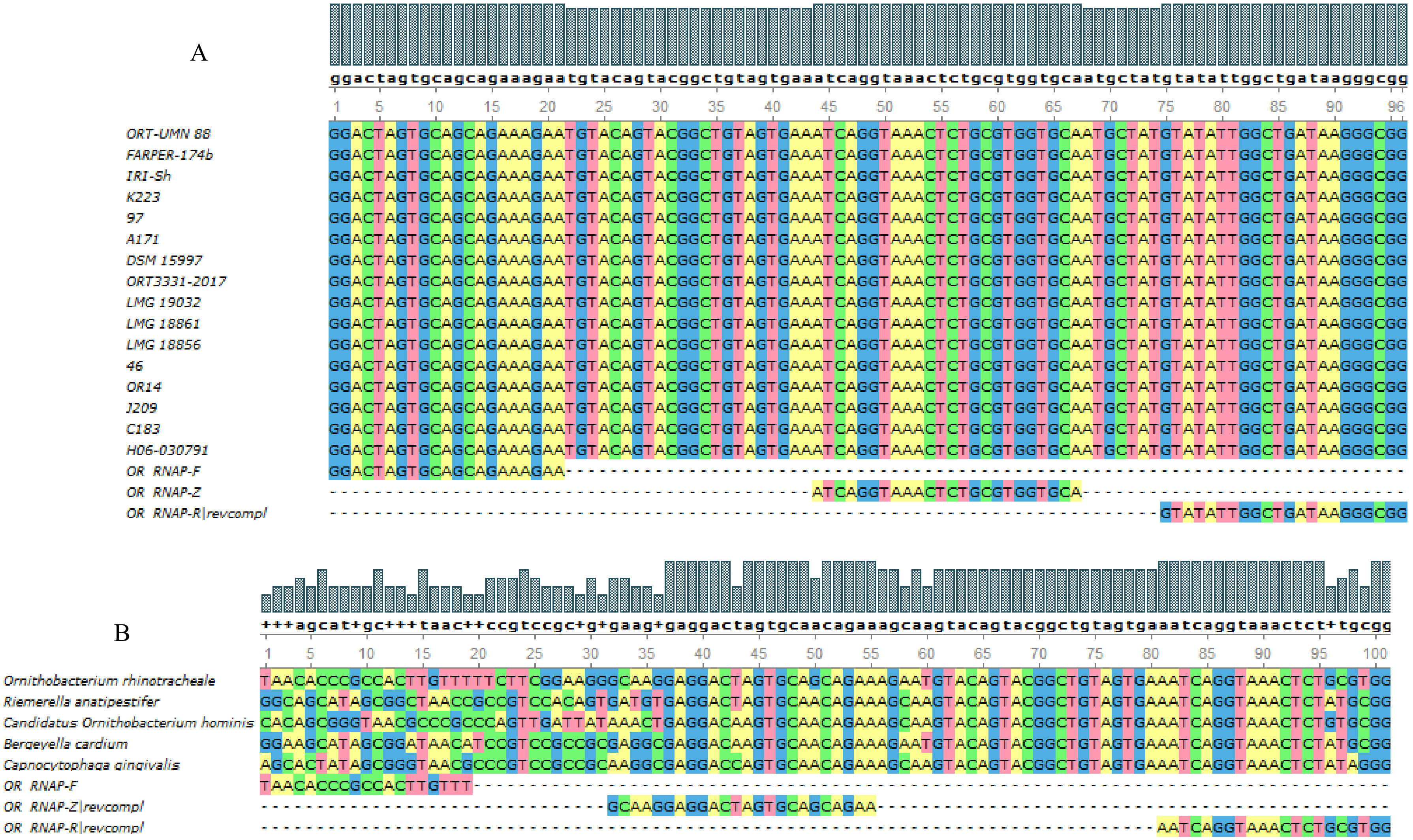

Figure S2: Multiple alignment of *rnaP* gene sequences in the region of annealing of primers and probe.
A) *O. rhinotracheale;* B) some related species.
The sequence of the reverse primer and probe is given in the reverse-complement orientation.

Table S5: Test results for clinical samples of *A. paragallinarum* (A.pg) (fragments of chicken beaks and sinuses).

Sample No.	Sample type	Mean *Ct* ± *SD*	*CV* (*Ct*), %	*A. paragallinarum* DNA
1	Known positive A.pg	18.28 ± 0.04	0.19	detected
2	Known positive A.pg	19.49 ± 0.22	1.12	detected
3	Known positive A.pg	20.49 ± 0.05	0.24	detected
4	Known positive A.pg	19.37 ± 0.11	0.55	detected
5	Known positive A.pg	19.29 ± 0.20	1.03	detected
6	Known positive A.pg	18.98 ± 0.02	0.11	detected
7	Known positive A.pg	19.43 ± 0.05	0.25	detected
8	Known positive A.pg	20.48 ± 0.25	1.24	detected
9	Known positive A.pg	20.24 ± 0.11	0.56	detected
10	Known positive A.pg	19.90 ± 0.16	0.78	detected
11	Known positive A.pg	19.61 ± 0.16	0.79	detected
12	Known positive A.pg	19.46 ± 0.14	0.73	detected
13	Known positive A.pg	20.37 ± 0.16	0.76	detected
14	Known positive A.pg	19.71 ± 0.08	0.43	detected
15	Known positive A.pg	20.59 ± 0.15	0.72	detected
16	Known positive A.pg	20.20 ± 0.16	0.81	detected
17	Known positive A.pg	19.46 ± 0.10	0.51	detected
18	Known positive A.pg	19.43 ± 0.16	0.84	detected
19	Known positive A.pg	20.00 ± 0.04	0.21	detected
20	Known positive A.pg	19.49 ± 0.06	0.29	detected
21	Known positive A.pg	19.43 ± 0.09	0.47	detected
22	Known positive A.pg	19.55 ± 0.18	0.94	detected
23	Known positive A.pg	19.50 ± 0.10	0.51	detected
24	Known positive A.pg	19.56 ± 0.08	0.40	detected
25	Known positive A.pg	20.36 ± 0.18	0.87	detected
26	Known positive A.pg	20.78 ± 0.19	0.92	detected
27	Known positive A.pg	19.62 ± 0.11	0.58	detected
28	Known positive A.pg	19.99 ± 0.03	0.14	detected
29	Known positive A.pg	20.79 ± 0.05	0.24	detected
30	Known positive A.pg	20.01 ± 0.11	0.53	detected
31	Known negative A.pg	–	–	not detected
32	Known negative A.pg	–	–	not detected
33	Known negative A.pg	–	–	not detected
34	Known negative A.pg	–	–	not detected
35	Known negative A.pg	–	–	not detected
36	Known negative A.pg	–	–	not detected
37	Known negative A.pg	–	–	not detected
38	Known negative A.pg	–	–	not detected
39	Known negative A.pg	–	–	not detected
40	Known negative A.pg	–	–	not detected
41	Known negative A.pg	–	–	not detected
42	Known negative A.pg	–	–	not detected
43	Known negative A.pg	–	–	not detected
44	Known negative A.pg	–	–	not detected
45	Known negative A.pg	–	–	not detected

Sample No.	Sample type	Mean *Ct* ± *SD*	*CV* (*Ct*), %	*A. paragallinarum* DNA
46	Known negative A.pg	–	–	not detected
47	Known negative A.pg	–	–	not detected
48	Known negative A.pg	–	–	not detected
49	Known negative A.pg	–	–	not detected
50	Known negative A.pg	–	–	not detected
51	Known negative A.pg	–	–	not detected
52	Known negative A.pg	–	–	not detected
53	Known negative A.pg	–	–	not detected
54	Known negative A.pg	–	–	not detected
55	Known negative A.pg	–	–	not detected
56	Known negative A.pg	–	–	not detected
57	Known negative A.pg	–	–	not detected
58	Known negative A.pg	–	–	not detected
59	Known negative A.pg	–	–	not detected
60	Known negative A.pg	–	–	not detected

Table S6: Test results for clinical samples of *O. rhinotracheale* (ORT) (chicken tracheal homogenate).

Sample No.	Sample type	Mean *Ct* ± *SD*	*CV* (*Ct*), %	*O.rhinotracheale* DNA
1	Known positive ORT	18.05 ± 0.10	0.55	detected
2	Known positive ORT	17.60 ± 0.21	1.17	detected
3	Known positive ORT	18.85 ± 0.17	0.9	detected
4	Known positive ORT	17.85 ± 0.20	1.11	detected
5	Known positive ORT	17.45 ± 0.19	1.09	detected
6	Known positive ORT	17.49 ± 0.26	1.50	detected
7	Known positive ORT	17.10 ± 0.15	0.87	detected
8	Known positive ORT	16.65 ± 0.19	1.15	detected
9	Known positive ORT	16.50 ± 0.13	0.81	detected
10	Known positive ORT	16.28 ± 0.21	1.26	detected
11	Known positive ORT	18.49 ± 0.06	0.31	detected
12	Known positive ORT	17.51 ± 0.12	0.69	detected
13	Known positive ORT	17.63 ± 0.30	1.68	detected
14	Known positive ORT	18.18 ± 0.16	0.89	detected
15	Known positive ORT	17.78 ± 0.25	1.43	detected
16	Known positive ORT	17.59 ± 0.23	1.29	detected
17	Known positive ORT	16.54 ± 0.13	0.77	detected
18	Known positive ORT	17.71 ± 0.11	0.6	detected
19	Known positive ORT	17.56 ± 0.16	0.89	detected
20	Known positive ORT	17.60 ± 0.23	1.29	detected
21	Known positive ORT	18.57 ± 0.36	1.94	detected
22	Known positive ORT	18.57 ± 0.16	0.84	detected
23	Known positive ORT	18.66 ± 0.12	0.64	detected
24	Known positive ORT	19.26 ± 0.16	0.84	detected
25	Known positive ORT	18.46 ± 0.07	0.38	detected
26	Known positive ORT	18.58 ± 0.50	2.70	detected
27	Known positive ORT	17.63 ± 0.26	1.48	detected

Sample No.	Sample type	Mean *Ct* ± *SD*	*CV* (*Ct*), %	*O.rhinotracheale* DNA
28	Known positive ORT	17.60 ± 0.19	1.09	detected
29	Known positive ORT	16.57 ± 0.17	1.02	detected
30	Known positive ORT	16.42 ± 0.30	1.81	detected
31	Known negative ORT	–	–	not detected
32	Known negative ORT	–	–	not detected
33	Known negative ORT	–	–	not detected
34	Known negative ORT	–	–	not detected
35	Known negative ORT	–	–	not detected
36	Known negative ORT	–	–	not detected
37	Known negative ORT	–	–	not detected
38	Known negative ORT	36.12	–	not detected [1]
39	Known negative ORT	–	–	not detected
40	Known negative ORT	35.24±0.49	–	not detected [2]
41	Known negative ORT	–	–	not detected
42	Known negative ORT	–	–	not detected
43	Known negative ORT	–	–	not detected
44	Known negative ORT	–	–	not detected
45	Known negative ORT	–	–	not detected
46	Known negative ORT	–	–	not detected
47	Known negative ORT	35.18	–	not detected [1]
48	Known negative ORT	–	–	not detected
49	Known negative ORT	–	–	not detected
50	Known negative ORT	–	–	not detected
51	Known negative ORT	–	–	not detected
52	Known negative ORT	–	–	not detected
53	Known negative ORT	–	–	not detected
54	Known negative ORT	–	–	not detected
55	Known negative ORT	35.37	–	not detected [1]
56	Known negative ORT	–	–	not detected
57	Known negative ORT	–	–	not detected
58	Known negative ORT	–	–	not detected
59	Known negative ORT	–	–	not detected
60	Known negative ORT	35.43	–	not detected [1]

1—the Ct is available in one replicate only.
2 —the Ct is above the cut-off limit equal to 32.
